# Creating 'good' self-managers?: Facilitating and governing an online self care skills training course

**DOI:** 10.1186/1472-6963-9-93

**Published:** 2009-06-08

**Authors:** Anne Kennedy, Anne Rogers, Caroline Sanders, Claire Gately, Victoria Lee

**Affiliations:** 1 National Primary Care Research and Development Centre, University of Manchester, Oxford Road, Manchester, M13 9PL, UK

## Abstract

**Background:**

In chronic disease management, patients are increasingly called upon to undertake a new role as lay tutors within self-management training programmes. The internet constitutes an increasingly significant healthcare setting and a key arena for self-management support and communication. This study evaluates how a new quasi-professional health workforce – volunteer tutors – engage, guide and attempt to manage people with long-term conditions in the ways of 'good' self-management within the context of an online self-management course.

**Methods:**

A qualitative analysis of postings to the discussion centre of 11 online classes (purposively selected from 27) run as part of the Expert Patients Programme. Facilitators (term for tutors online) and participants posted questions, comments and solutions related to self-management of long-term conditions; these were subjected to a textual and discursive analysis to explore:

a) how facilitators, through the internet, engaged participants in issues related to self-management;

b) how participants responded to and interacted with facilitators.

**Results:**

Emergent themes included: techniques and mechanisms used to engage people with self-management; the process facilitators followed – 'sharing', 'modelling' and 'confirming'; and the emergence of a policing role regarding online disclosure. Whilst exchanging medical advice was discouraged, facilitators often professed to understand and give advice on psychological aspects of behaviour.

**Conclusion:**

The study gave an insight into the roles tutors adopt – one being their ability to 'police' subjective management of long-term conditions and another being to attempt to enhance the psychological capabilities of participants.

## Background

Healthcare settings are becoming evermore varied and boundaries of expertise are shifting alongside contemporary cultural and policy changes. In chronic disease management, patients are now cast as 'experts' increasingly called upon to undertake a new quasi-professional role as lay tutors within self-management training programmes. [1] This section of the health-care workforce is valued not for any medical training they may possess but because of their experience of living with a long-term condition. Access to self-management programmes for high numbers of people with long-term conditions has been made possible because of a large volunteer workforce of trained tutors; this group is the focus of this paper.

In particular, we are interested in how individuals tasked with running an online self care support programme attempt to achieve the overarching policy aims of engaging and managing people with long-term conditions in self-management.[2] The internet constitutes an increasingly significant interactive healthcare setting and is increasingly becoming a key arena for self-management support and communication.[3]

There are tensions in using lay people to teach others how to self-manage which relate to the value placed on their expertise and ability and on the limits of what they are able to teach. Prior [4] attempts to draw a boundary around the expertise domains of patients and health professionals; lay knowledge and expertise is concerned with the experiential which means it is invariably limited, idiosyncratic and generally based on one case. Prior argues that for the most part, lay people are not experts as they are unskilled in medical fact gathering or diagnosis (the domain of the health professional); in other words, lay people can be wrong. However, there is consensus that lay people can be deemed to be experts in the day-to-day experience of living with a long-term condition.

The training of Expert Patients Programme (EPP) tutors is focussed on ensuring that tutors learn to deliver the course 'by the book' in a structured manner.[5] This method of training is used to ensure quality control of the courses and is viewed as providing a safe way for lay people to deliver health education.[6] A national survey of EPP tutors found that whilst the majority felt the training was a good use of their time, a significant number wanted additional training in group management skills and dealing with challenging participants. [5]

A review of lay-led self-management [7] found that the literature is represented mainly by the work of Lorig and colleagues who contend that as their research shows no significant differences in patient outcomes between lay-led and non lay-led approaches (ie professionally led or mail delivered), then financial benefits favour a lay-led approach. [8-10] However, Taylor and Bury argue that there has not been enough comparative research to justify claims that self-management courses should be lay-led.[11]

The term 'peer' education is mostly used to the field of health promotion in relation to sexual health, smoking and drug use; whereas 'lay' education is more frequently associated with self-management of long-term conditions. Most research has concentrated on lay or peer educators' experiences or the processes of implementing initiatives.[12-16] Such work has found that lay people, although initially apprehensive, generally enjoy the experience and gain personally from it. Larkey et al[17] studied communication strategies used by peer educators in a worksite intervention designed to change dietary habits. Their analysis outlined ways in which peer educators used social influence to change behaviour including: teasing; mock competition; role modelling; giving material; creating context; foot-in-the-door; encouragement; and responding to needs. There were gender and cultural differences in the strategies used and different strategies were used in group or individual contexts.

Whilst there is much research on the way professionals communicate within consultations, less is known about how lay people tasked with providing health education set about the process. A qualitative evaluation of the online version of the EPP allowed us access to the online content of discussion centres where facilitators (the term for online course leaders, community leaders are called tutors) and participants were able to post questions, comments and solutions related to self-management of long-term conditions. The following analysis aimed to:

• Explore how facilitators engaged participants in issues related to self-management.

• Explore how facilitators viewed their role and the effect this had on the way they interacted with participants.

This work forms part of a larger, national evaluation of the EPP and this analysis is set in the context of our published findings. [1,5,18-26] Additional file 1[27] gives details of the course content and Additional file 2 describes the training for the online facilitators. EPP tutors and facilitators are managed by a system of quality assurance to ensure standardised delivery of the courses. [1,6]

## Methods

The EPP Online course was run as a joint venture between Stanford University in the USA and the UK Department of Health and the quantitative results have been published separately.[28] Researchers at the National Primary Care Research and Development Centre were commissioned to undertake a qualitative evaluation of the online course. All course participants gave informed consent, were aware they were taking part in a closely monitored pilot scheme and that researchers would have access to their online postings. Ethical approval was obtained from an NHS ethics committee (Northern and Yorkshire MREC 05/MRE03/31) and Stanford University Institutional Review Board.

EPP Online was run between July 2005 and September 2006. As part of the qualitative research process, 11 out of the 27 classes completed during the trial period were purposively selected for analysis of postings to the difficult emotions and problem solving sections of the discussion centre as these were the sections where there was most interaction between the participants and facilitators. Selection was based on the classes which had most participants with the highest or lowest baseline characteristics (see table 1)

**Table 1:** Purposeful selection of classes to be included in the analysis

**Class ID**	**Class characteristic**	**Class characteristic**	**Class characteristic**	**Class characteristic**
43	Highest (n) participants	Lowest (n) retired		
44	Highest (n) participants	Low self-reported health		
59	Lowest mean age	Lowest (n) housework^1^		
74	Highest (n) retired	Highest (n) disabled	Highest (n) lung conditions	
64	Highest (n) Diabetes	Highest (n) hypertension	Highest (n) mental health conditions	Highest (n) heart conditions
66	Lowest (n) participants	Highest (n) COPD	Highest (n) cancer	
67	Highest self-reported health	Lowest (n) arthritis		
72	Highest (n) Diabetes	Lowest (n) asthma		
73	Lowest (n) males	Lowest (n) mental health conditions		
62	Highest with degree or professional qualification	Highest (n) males	Highest (n) full-time employed	Lowest (n) Disabled
68	Lowest (n) white ethnicity			

(n) = number of

^1 ^Actively working in home environment (e.g. housework, caring for family)

Data from the postings to each class by participants and facilitators were entered into an Access database and codes and comments created within Atlas.ti. The entries from the discussion centres for all respondents (participants and facilitators) were merged into a text file for each weekly session for each class. This allowed a narrative reading of the classes to give an overview of how the facilitators dealt with queries and problems and to pull out patterns of responses. A coding framework was then created relating to the specific posts of the facilitators and the analysis focussed on emerging themes related to engaging participants in self-management. Whilst only the postings from the facilitators have been included and considered in this analysis, these are set within the narrative context of each class.

All the quotes are presented as they were typed by participants; we have not corrected typos or spellings.

## Results

**Figure 1:**
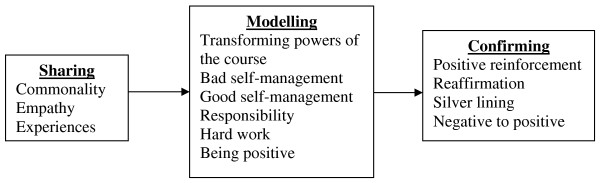
**Model to show the process of engaging participants with self-management**.

The facilitators used a number of techniques to engage people with self-management. The way the facilitators worked followed a process of sharing, modelling and confirming (see figure 1: Model to show the process of engaging participants with self-management).

### Sharing

Facilitators built on the input of the group to dispel fear and isolation associated with living and managing long-term conditions by emphasising the commonality of problems and feelings and trying to get participants to say 'Yes, I feel that too'. This was an attempt to develop a groundswell of belief that action was possible – by implicating that there were others out there with the same symptoms and feelings who were able to cope.

'I was very surprised when I learnt that most of us with chronic conditions suffer from this unpredictability, I had thought it was only those with my condition. It's a big help to me to know this, I then think just how many thousands of us are having this problem, and most are managing well, and then, so can I!' (class 73)

Facilitators posted supportive and empathic responses which implied that they had the same feelings and suffered in a similar way to the person who posted the problem. This seemed to be being done to ensure that participants felt the course leaders possessed the necessary personal experience and understanding of what they themselves were experiencing. There was an emphasis on giving voice to such experiences:

'Please feel free to share your exasperations etc. Hope your stress starts to decrease a little; as you say, it's what none of us need.' (class 43)

There was general encouragement to share rather than contain experiences and facilitators quite often depicted themselves as being vulnerable and weak as a means of trying to relate to and engage with participants.

'I sympathise – sometimes my arthritis makes me go up and down stairs on my back side-it is frustrating – the only thing to do is to take it slowly, we'll get there in the end. take care' (class 73)

Sharing of experiences also encompassed the day-to-day difficulties, inconveniences and suffering of living with a long-term condition. The sharing and relating of experiences was often done in a jokey; 'clubby'; 'we're all in this together' way.

'Hi X! i too know what you mean with regard to medications!! The way I cope is to think about what my life would be like without the meds!! I couldnt cope at all!! For me it is just a necessary evil...try to focus on the postive aspects of taking the meds, what it enables you to do. I hope this helps! Good luck x' (class 59)

### Modelling

Being a role model is a strategy EPP course leaders are taught to enhance the self-efficacy of course participants.[29] This strategy was used in a variety of ways in the online version.

On many occasions, modelling was used to showcase the benefits of the EPP itself. There was a detectable 'evangelical' edge to much of the advice given by the facilitators. This has been noted in other work related to the role of self care skills trainers,[1,30] in particular the way policy rhetoric and the enthusiasm of some course participants have been combined to portray the programme as a life changing event in order to promote and sell the course to participants and health care organisations. Participants were encouraged to use the strength of the techniques introduced on the course to 'transform' themselves. Facilitators gave examples of how the course had changed their own behaviour and attitude.

'My life just wouldn't work without the Action Planning ..has become a habit – and I have used Self Talk so many times now, telling myself what is good for me – that I now believe it. Hug,' (class 64)

**Figure 2:**
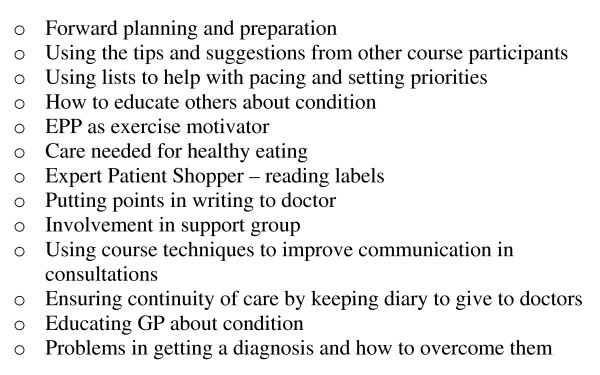
**Examples of good self-management modelled by tutors on class 64**.

Throughout every class, facilitators modelled examples of what was viewed as 'good' self-management (see figure 2: Examples of good self-management modelled by tutors on class 64). However, facilitators sometimes turned this strategy on its head and modelled (in a confessional manner) how they used to behave before they learned how to be an exemplary self-manager (inevitably as a result of going on an EPP course).

'In my experience, I was drowning in anger, and negativity, and realised that I was destroying myself – the real me – far more effectively than any condition... Then I discovered that I could channel some of the anger into a kind of stubborness, and use it to find a different way of doing what I wanted. ... A major part of this process was the luck of being able to talk things over with people who really understood because they had been through it themselves, and were willing to support and mentor me...even when I was being a complete pain. I met these people when attending an EPP community course as a participant nearly four years ago, and some of them are still friends.' (class 44)

There was an emphasis on the need to be a 'responsible' self-manager and on what Frank has referred to as the hard work of being successfully ill.[31] Facilitators gave examples to depict this; however, they also added caveats such as the limitations placed on them by their symptoms flaring up.

*'it is not easy to think of exercising when living with fatigue and breathlessness, and when I am having similar problems the last thing I want to do is exercise. I know, though, that the right sort of exercise will help with my symptoms in the long run, and help prevent problems in the future, so I do chair exercises, which I find that I can manage on all except my very worst days.' *(class 72)

A great deal of modelling and advice drew on the concept of gaining and retaining a positive attitude.

'Such a good positive feeling being able to say you are comfortable in the path your life has taken. Use that positivity to work for you and am sure you will be pleasantly surprised where the path takes you. Confidence builds self esteem and that is something we all need loads of. Keep smiling as you use far less muscles to smile than you do to frown.' (class 68)

### Confirming

A final identifiable aspect of engagement and management used by the facilitators was to encourage people to continue on their journey of transformation into becoming 'expert patients'. This was done by getting people to recognise how far they had come already through using a lot of praising and overtly positive language (eg wow, brilliant, you're a star). Even small indications of thoughts or actions were framed as being steps on the road to good self-management *'You recognise some of the problem, which is the first step in problem solving.' (class 68)*

Facilitators made a point of congratulating the participants on all their achievements and held up certain posts which emphasised free will and choice as good examples of self-management. Such comments positively reinforced self-management actions and participants were encouraged to be proud of their success and for being responsible citizens.

'It shows that you have chosen to actively manage your condition – you are an example to us all, thank you.' (class 66)

The facilitators attempted to demonstrate how people could reconstruct their stories so as to emphasise positive ways lives are changed by having a long-term condition (a silver lining ethos) such as new skills learnt and gain in terms of strength of character (becoming a better person).

'I always try to think of the fact I know I have grown as a person because of my condition and know that without my condition and the things I can't do now I would be a different person and have a lot less skills than I do have now. I think that makes sense, but anyway I suppose I'm saying I think I understand and I think I'm a better person because of my condition' (class 67)

Part of the process implied another type of reconstruction – that of changing seemingly negative and depressing incidents or thoughts into positives, a standard technique used in cognitive behavioural therapy. These aspects of quasi positive psychology were introduced during the 'self-talk' section of the course where participants were encouraged to change the way they talked and thought about problems and emotions. The second quote illustrates the difficulties for facilitators in achieving this online, the facilitator has stepped in with a solution rather than leaving the individual to think through the changes needed.

'I was asked a question once, name a time when worry or guilt has helped you. It started me thinking that they are useless activities that sap energy and leave us feeling negative. Perhaps you can turn your guilt into feelings of satisfaction that you are looking after yourself and your needs and whatever is was can be done another day, I'm still working on this, hope it helps.' (class 62)

'you are not a hypochondriac – You are experienced in living with pain.' (class 64)

### Role tensions and conflicts

The analysis found a number of areas where facilitators exhibited contradictory approaches to a particular issue.

Throughout the course, facilitators placed an emphasis on the commonality between people with long-term conditions using phrases such as: '*you are not on your own'; 'I've got that t-shirt too'*. However, people came on the course because of their long-term medical condition and often posted condition *specific *queries. Facilitators' general response to such queries was to block them ('the course is not designed to deal with medical problems') and requested the participants not to give medical advice to each other (contra to peoples' wishes and expectations). However, in practice, facilitators often contradicted their 'commonality' generic approach and adopted an 'everyone is different' approach with advice to seek medical help.

'Although it looks from your post as if we might have the same condition, everyones experiences and outcomes really are so completely different in this that you might have half a dozen women all telling you earnestly that theirs is the only way to go, but it is your health professionals who know your medical history, and you know your own body and how it feels.' (class 44)

An anonymous online course allowed people to be open about their problems or opinions. However, facilitators appeared to be sensitive to the risks of allowing a free for all and were concerned that certain opinions or approaches to life might prove upsetting. So the facilitator's role was both to enhance and constrain interactions in ways which conformed to what was viewed as appropriate. In actuality, there were very few examples of facilitators having to actively 'police' and constrain postings although facilitators were alert for any disrespectful posts.

'Respect for oneself and each other is paramount if we all are to benefit from the experiences we share on this course. Its just a question of thinking before you speak..we all have a right to express an opinion but we should be mindful of the effect these could have on others and respect everyones right to and opinion but not to use that opinon to jeer or judge another participant.' (class 44)

One aspect of the policing role was the license to comment on participants' behaviour in ways which were not motivating or enabling but which adopted a moral tone about expectations of normal good behaviour.

'I'm sure you are fully aware that comfort eating does exactly the opposite to what we would like to happen' (class 67)

The facilitators were influenced by policy statements concerning the role of self-management in reducing the burden of long-term condition management on the NHS. They were keen to extol self-management as the potential saviour of the NHS in that it saved money by keeping people healthy and out of the system. The following quote also exemplifies how facilitators expound the win win aspects of self-management because as well as helping the NHS there are potential rewards for the individual in focussing on their own worth.

'I am on my way to become a great self-manager and am a much happier person – and in the process ..I can only imagine how much this is saving the NHS in time and money too! Look after yourself – YOU are worth it!:)' (class 74)

The converse to this was that facilitators also felt their role was to act as consumer champions and encourage people's 'right' to use NHS services, for example in seeking a second opinion. Signposting people to their GP was a frequent response to queries of a medical nature.

'If you have any 'out of the usual' symptoms the best thing is to go to your GP. I'd go back every few days, at least then it is being noted how you are and how your problem is manifesting itself. I'd also ask to be referred back to your specialist...after all they know the best about the course of your condition' (class 73)

The facilitators seemed to work on the implicit rule that giving medical advice was not 'allowed' and did their best to suppress any attempts to do this. It did, however, appear perfectly legitimate to give psychological advice and many facilitators seemed happy to adopt a quasi counsellor role. Certainly, the stated intent of the course developers was to change people's behaviour and attitudes by increasing individual levels of self-efficacy and the notion of promoting individual change seemed to segue fairly naturally into lay interpretations of the language of psychotherapy.

'Would it be a good idea to begin to tackle this in a neutral environment where you are both relaxed?' (class 73)

A section of the course focuses on making advanced directives. Participants are asked to consider why they have not made advanced preparations for health care or why this is difficult to think about. Previous research has demonstrated that issues raised in the session can disrupt some aspects of illness adaptation and existing views about death and dying.[23] This was a demanding session for facilitators who had to develop strategies to deal with responses – or, more usually – lack of responses. During training, facilitators were provided with certain replies to deal with frequently occurring situations or problems and many facilitators had a stock reply for this section which seemed to come from a belief that every aspect of the EPP course was important and the following quote is a reply used by one facilitator (with slight variations) as a response to most participants in the class:

'Please try to think of this as just another part of self-management, this time for the future rather than now.' (class 43)

Other facilitators recognised that the session was problematical and probably did not fit well with the rest of the course content. One or two felt strong enough in their convictions to be openly questioning about the rationale for including 'Living Wills' and were honest in admitting difficulties in moderating the discussions that it generated.

'The course just gives a very brief overview on the subject, but I will forward your comments to Stanford and should they wish to respond I will feed back to you. This subject can evoke quite heated debates but perhaps this is not the forum for that, as I for one donot have the legal background to comment on profound statements.' (class 68)

## Discussion

Access to the interactions of facilitators and participants on online EPP courses has illuminated the techniques and process used by lay leaders to turn people with long-term conditions into socially responsible self-managers. The analysis presented here was limited to assessing the methods and approaches online facilitators used to engage participants with self-management; the views and outcomes of participants will be the subject of different analyses.

The techniques used followed a pathway of sharing, modelling and confirming. Community tutors selected to be online course facilitators were considered by EPP managers (and by themselves) to be those most experienced in delivering the community version of EPP and strong advocates of the course.

The idea of a state-endorsed self-management course led by volunteer lay-leaders (the EPP) has come to seem very attractive to policy makers.[11] The political drive to increase people's responsibility for management of their conditions fits with the sociological concept of 'ethopolitics' – the politics of life itself and how it should be lived as proposed by Rose.[32] Rose proposes that, in western democracies, our personalities, subjectivities and relationships are not private matters but are intensively governed by state bureaucracies, policies and initiatives.[33] In the late 20^th ^century, a new group of professionals has developed with expertise in subjectivity who claim to 'understand the psychological aspects of the person and to act upon them or to advise others what to do' (page 3). He calls this group 'engineers of the human soul'. It could be argued that volunteer EPP tutors have been given dispensation and power from the state to mould and guide people with long-term conditions in ways to become better self-managers.

The 'online' policing role adopted by facilitators was characterised by two aspects: checking the acceptability of the postings; and a more over-arching aim to promote responsible citizenship. Personal examples of the difficulties of living with a long-term condition were used as opportunities to sell the EPP to show how the course could be utilized to change attitudes and behaviour. The moral tone that facilitators sometimes resorted to was both an indication of lack of exposure to the theories and methods used by adult educationalists (where the role of the educator is to enable and support the pupil to learn for themselves) as well as a mark of the strong need the facilitators felt to be advocates for the EPP. In this respect tutors are no different to health professionals struggling to engage with supporting peoples' efforts to self-manage. In previous research we have shown that there is a need for education to equip professionals with such techniques to work effectively with patients dealing with the longer-term effects of chronic illness.[34] Being strong advocates meant they defended the course content even when it at times caused obvious upset, such as during the session on advanced directives.

Facilitators were keen to increase participants' responsibility for self-managing and often professed to understand and give advice on psychological aspects of behaviour. However, the exchange of what was seen as medical advice amongst the participants was discouraged. The EPP course is firmly rooted in the psychological concept that improvements in self-efficacy will lead to changes in behaviour[29] – this is the main self-management technique taught on the course (through goal setting and action planning). Facilitators also drew on other psychological approaches, for example encouraging participants to reconstrue how they viewed themselves in the 'self-talk' section and turning negative views of life into positive thoughts and actions. They did this by modeling their own previously 'bad' behaviour and by confirming responses that concurred with a model of positive transformational change aka Prochaska.[35] It does appear that in this aspect, facilitators were going beyond the boundary suggested by Prior[4] of being experts in the experience of living with a long-term condition and stepping into the more psychological role of a counselor. The quasi psychological approach used by facilitators equates with aspects of the positive psychology movement. We know from previous research with the EPP that this approach is frequently seen as beneficial by participants. Nonetheless it is clear that such an ethos may also be vulnerable to the critique of such an approach[36] which suggests that such an approach can start to form a type of tyranny in emphasizing the positive advantages of having positive thoughts and attitudes because those who may find it difficult to transcend pain and the difficulties associated with having a long-term condition may find such an approach adds 'insult to injury'. The selective recruitment and drop out rates of those attending the EPP may be indicative of this.[22] Additionally, we have found indications of resistance to this ethos from course participants.[23]

Responses and posts from the facilitators were consistently very positive and encouraging. An analysis of language used in online support groups, found that women in particular tend to use superlatives and express strong feelings.[37] Bar-Lev [38] contends that emotional discourse online aims to produce intimacy between strangers and impose a moral dimension to the dynamics of exchanges.

These findings are likely to be applicable to lay tutors on community courses as any differences between the community and online course seem to be related to the intensity and depth of response (more apparent online) rather than techniques of engaging people with self-management.

## Conclusion

This study provides insights into the roles on-line lay tutors adopt – one being their ability to 'police' subjective management of long-term conditions and another being to attempt to enhance the psychological capabilities of participants. The generic nature of the course allowed the sharing of common experiences, but led to certain tensions for the facilitators, particularly around participants' expectations of getting medical advice. The reliance on volunteer tutors to teach self-management skills is attractive to policy makers, but those who select, manage and train volunteers for such posts need to be aware of the tensions resulting from delivering a highly structured course to people with diverse and unknown needs and consider whether lay tutors require additional training to gain skills in techniques to encourage behavioural change.

## Competing interests

The authors declare that they have no competing interests.

## Authors' contributions

AK carried out the initial analysis of the raw data from the postings to the online course and drafted the manuscript. AR assisted with the analysis and contributed to the manuscript. CS read the raw data and confirmed the emerging themes. CG and VL interviewed the facilitators and contributed to discussions around the themes. All authors participated in the design and co-ordination of the study and approved the final manuscript.

## Pre-publication history

The pre-publication history for this paper can be accessed here:



## Supplementary Material

Additional file 1 **Box 1 – Details of EPP online and community courses**. Written description of the format and content of the EPP courses. Click here for file

Additional file 2 **Box 2 – details of additional training for online facilitators**. Bullet point description of the objectives of the training course. Click here for file
