# Supplementary material for: Creating 'good' self-managers?: Facilitating and governing an online self care skills training course
Source: BMC Health Serv Res. 2009 Jun 8;9:93. doi: 10.1186/1472-6963-9-93 (PMC2699343; doi:10.1186/1472-6963-9-93)
Supplement: Additional file 1 — Box 1 – Details of EPP online and community courses. Written description of the format and content of the EPP courses. [file 1472-6963-9-93-S1.doc]

**Box 1 – Details of EPP online and community courses**

The EPP is an anglicised version of the Chronic Disease Self-management Program developed by researchers at Stanford University in the USA and consists of six sessions held on a weekly basis. The topics covered include: relaxation, diet, exercise, fatigue, breaking the symptom cycle, managing pain and medication, and communication with health care professionals. In the community course, these topics are introduced by the course leaders who follow a written script, in the online version of EPP, topics are covered through written text. In the community, groups are made up of 10 to 16 people with a long-term condition who have self-referred to the course. Online courses cover the same topics each week but each course can have up to 25 participants. Volunteer community course leaders are called tutors and online course leaders (who require additional training) are called facilitators. EPP course leaders are taught to act as ‘role models’. Goal setting and action planning sessions form a key part of the training course and each leader and participant is expected to set out a goal with specific actions they plan to undertake during the coming week. This activity is intended to increase self-efficacy as participants are taught how to refine their plans until they are confident they will be able to achieve them; the following week they report on their success and the group ‘problem solves’ any difficulties encountered. ‘Problem solving’ entails asking if the individual has any ideas to solve the problem, then asking the group for possible solutions. In the online course, such interactive discussion takes place in the discussion centre and participants are prompted by the course facilitators to a) post a problem they have related to the week’s topic and b) give responses to other participants who post problems. If no participants respond to a post, then the facilitators are expected to write a helpful response. The specific ‘problem’ topics introduced each week to be ‘solved’ through posts from participants are:

- Exercise barriers
- Healthy eating barriers
- Barriers to advanced planning (making a Living Will)
- Communication barriers
- Problems with health care providers
