# Supplementary material for: Creating 'good' self-managers?: Facilitating and governing an online self care skills training course
Source: BMC Health Serv Res. 2009 Jun 8;9:93. doi: 10.1186/1472-6963-9-93 (PMC2699343; doi:10.1186/1472-6963-9-93)
Supplement: Additional file 2 — Box 2 – details of additional training for online facilitators. Bullet point description of the objectives of the training course. [file 1472-6963-9-93-S2.doc]

Box 2 – details of additional training for online facilitators

Before they were able to deliver the online course, potential facilitators:

Took part in an online EPP course as participants

Attended a 2-day residential course with the following objectives:*

At the end of the Facilitator’s Training Event, participants will be able to:

Facilitate the EPP Online course.

Utilise the Self-Management of Long-term Health Conditions handbook and the Facilitator’s Manual.

Successfully complete all daily and weekly tasks required of an EPP Online facilitator.

Handle problems, which arise in online group situations.

Feedback information about both the content and the process of the course to EPP Online Project Staff and Stanford Staff.

Work with other Facilitators for assistance as necessary and appropriate.

Co-operate with EPP Online Project Staff and Stanford University Staff in a professional manner.

Understand the background to EPP Online and the Pilot Project.

All volunteers to gain knowledge of the administration of the course, their promotional role, research, and budget restraints of the EPP Online Pilot Project.

Understand the process of becoming accredited Online Facilitators.

*Sourced from training material produced for a training event
